# Efficacy of Once-Weekly Semaglutide vs Empagliflozin Added to Metformin in Type 2 Diabetes: Patient-Level Meta-analysis

**DOI:** 10.1210/clinem/dgaa577

**Published:** 2020-08-22

**Authors:** Ildiko Lingvay, Matthew S Capehorn, Andrei-Mircea Catarig, Pierre Johansen, Jack Lawson, Anna Sandberg, Robert Shaw, Abby Paine

**Affiliations:** 1 University of Texas Southwestern Medical Center at Dallas, Dallas, Texas; 2 Rotherham Institute for Obesity, Clifton Medical Centre, Doncaster Gate, Rotherham, UK; 3 Novo Nordisk, Vandtårnsvej, Søborg, Denmark; 4 Zedediah Consulting on behalf of DRG Abacus (part of Clarivate), Wokingham, UK

**Keywords:** indirect comparison, individual patient data, GLP-1 receptor agonist, SGLT-2 inhibitor, type 2 diabetes

## Abstract

**Context:**

No head-to-head trials have directly compared once-weekly (OW) semaglutide, a human glucagon-like peptide-1 analog, with empagliflozin, a sodium–glucose co-transporter-2 inhibitor, in type 2 diabetes (T2D).

**Objective:**

We indirectly compared the efficacy of OW semaglutide 1 mg vs once-daily (OD) empagliflozin 25 mg in patients with T2D inadequately controlled on metformin monotherapy, using individual patient data (IPD) and meta-regression methodology.

**Design, Setting, Participants, and Interventions:**

IPD for patients with T2D receiving metformin monotherapy and randomized to OW semaglutide 1 mg (SUSTAIN 2, 3, 8 trials), or to OD empagliflozin 25 mg (PIONEER 2 trial) were included. Meta-regression analyses were adjusted for potential prognostic factors and effect modifiers.

**Main Outcome Measures:**

The primary efficacy outcomes were change from baseline to end-of-treatment (~1 year) in HbA_1c_ (%-point) and body weight (kg). Responder outcomes and other clinically relevant efficacy measures were analyzed.

**Results:**

Baseline characteristics were similar between OW semaglutide (n = 995) and empagliflozin (n = 410). Our analyses showed that OW semaglutide significantly reduced mean HbA_1c_ and body weight vs empagliflozin (estimated treatment difference: −0.61%-point [95% confidence interval (CI): −0.72; −0.49] and −1.65 kg [95% CI: −2.22; −1.08], respectively; both *P* < 0.0001). Complementary analyses supported the robustness of these results. A significantly greater proportion of patients on OW semaglutide vs empagliflozin also achieved HbA_1c_ targets and weight-loss responses.

**Conclusions:**

This indirect comparison suggests that OW semaglutide 1 mg provides superior reductions in HbA_1c_ and body weight vs OD empagliflozin 25 mg in patients with T2D when added to metformin monotherapy.

Glucagon-like peptide-1 receptor agonists (GLP-1RAs) and sodium–glucose co-transporter-2 inhibitors (SGLT-2is) are established therapies for type 2 diabetes (T2D) (1). Guidelines on hyperglycemic management recommend either GLP-1RAs or SGLT-2is as second-line treatments for patients with T2D on metformin monotherapy (1, 2), or as first-line treatments in patients with T2D at high or very high cardiovascular risk (3). Comparative efficacy analyses of GLP-1RAs and SGLT-2is could aid physicians in choosing between these 2 classes, particularly because they are similarly positioned in the American Diabetes Association−European Association for the Study of Diabetes Consensus report treatment algorithm for T2D (1).

Of the 4 US Food and Drug Administration–approved SGLT-2is (canagliflozin (4), dapagliflozin (5), empagliflozin (6), and ertugliflozin (7)), only 2 have been compared directly with GLP-1RAs in randomized controlled trials (RCTs): canagliflozin with once-weekly (OW) subcutaneous semaglutide in SUSTAIN 8 (8), and empagliflozin with oral semaglutide in PIONEER 2 (9). Given that there are so few RCTs directly comparing the efficacy of these 2 treatment classes, indirect comparisons of treatments that have not been evaluated in a head-to-head trial can provide valuable and much needed additional insight into this clinically important knowledge gap.

Various indirect treatment comparison methods have different properties that confer advantages and disadvantages. For example, network meta-analyses (NMAs) provide an important and robust method of making indirect comparisons between treatments using all available published data collected in a systematic manner and preserving randomization (10). However, when individual patient data (IPD) are available, meta-regression analyses of these data present another valid option for indirect treatment comparisons (Fig. 1) (11). In IPD meta-regression analyses, potential prognostic factors and effect modifiers can be adjusted at an individual patient level, allowing for a better isolation of the effect of a single factor (treatment) on an outcome of interest than might be achieved with published aggregate data, thereby enabling a potentially less-biased comparison between trials (12). In addition, clinically relevant outcomes, including changes in lipid profile, postprandial plasma glucose, and estimated glomerular filtration rate (eGFR), may differ in how they are reported across published aggregate data, or may not be reported at all. Therefore, these outcomes cannot always be analyzed with NMA methodology; to address this, IPD meta-regression analyses can be used.

**Figure 1.**
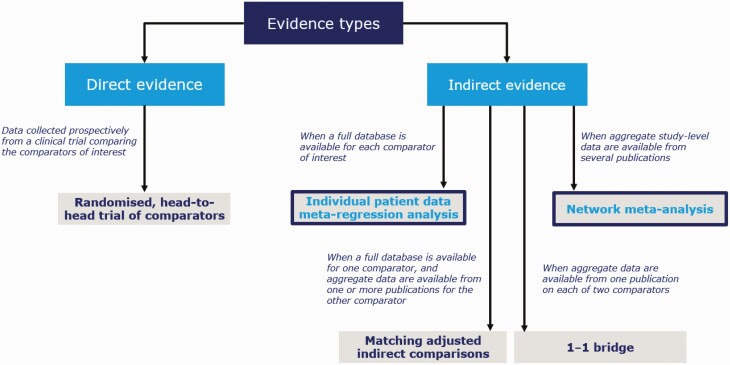
Standard of evidence for some examples of direct and indirect comparisons (10, 12).

Indirect comparisons of OW semaglutide vs SGLT-2is on efficacy outcomes have been previously reported, including the systematic literature review and NMA by Sharma et al. (13). The authors analyzed published aggregate data and concluded that OW semaglutide 1 mg was significantly more effective than empagliflozin, dapagliflozin, and canagliflozin in lowering glycated hemoglobin (HbA_1c_) and reducing body weight in patients with T2D that is inadequately controlled with metformin monotherapy (13). However, with the availability of IPD for OW semaglutide 1 mg (from SUSTAIN) and once-daily (OD) empagliflozin 25 mg (from PIONEER), an IPD meta-regression analysis is possible.

The aim of our analysis was to compare indirectly the efficacy of OW semaglutide 1 mg vs OD empagliflozin 25 mg over a 52-week period in patients with T2D inadequately controlled on metformin monotherapy, using IPD and meta-regression methodology. We assessed common measures of efficacy, such as change in HbA_1c_ and body weight, as well as additional endpoints that are clinically relevant but could not previously be indirectly assessed in other studies (eg, lipid parameters and eGFR) because of absence of available data.

## Materials and Methods

### Population data included from the SUSTAIN and PIONEER trials

IPD for all patients with T2D receiving background metformin monotherapy and randomized for 52 or 56 weeks to OW semaglutide 1 mg (SUSTAIN 2, 3, and 8), or to OD empagliflozin 25 mg (PIONEER 2) were included in this IPD meta-regression analysis. The trial designs and patient selection criteria have been published previously (8, 9, 14, 15); Table 1 provides a brief summary of each trial. This analysis was performed as an unanchored IPD meta-regression analysis because a common comparator (an identical treatment group across RCTs) was not evaluated in the included trials. In both SUSTAIN 8 and PIONEER 2, a criterion for inclusion was that patients had to be receiving metformin monotherapy (8, 9), so in these trials, all patients in the OW semaglutide and OD empagliflozin arms were included. Patients in SUSTAIN 2 and 3 who were on background therapies other than metformin monotherapy were excluded.

**Table 1.** Overview of the Design of the Trials Included in the IPD MR Analysis

Trial Characteristics	SUSTAIN 2 (15)	SUSTAIN 3 (14)	SUSTAIN 8 (8)	PIONEER 2 (9)
Treatment arm included in this analysis	OW s.c. semaglutide 1 mg	OW s.c. semaglutide 1 mg	OW s.c. semaglutide 1 mg	OD oral empagliflozin 25 mg
Other treatment arms	OW s.c. semaglutide 0.5 mg Sitagliptin 100 mg	Exenatide extended release 2 mg	Canagliflozin 300 mg	Oral semaglutide 14 mg
Randomization	Randomized	Randomized	Randomized	Randomized
Blinding	Double-blind	Open-label	Double-blind	Open-label
Duration, weeks	56	56	52	52
Baseline HbA_1c_ inclusion criteria	7.0−10.5% inclusive	7.0−10.5% inclusive	7.0−10.5% inclusive	7.0−10.5% inclusive
Background medications	Stable daily dose of metformin, pioglitazone, or rosiglitazone, OR a combination of metformin + pioglitazone, OR a combination of metformin + rosiglitazone	Receiving stable daily dose of 1 or 2 OADs (metformin and/or thiazolidinediones and/or sulfonylureas)	Stable daily dose of metformin	Stable daily dose of metformin
Primary endpoint	CFB to 56 weeks in HbA_1c_	CFB to 56 weeks in HbA_1c_	CFB to 52 weeks in HbA_1c_	CFB to 26 weeks in HbA_1c_*
Confirmatory secondary endpoint	CFB to 56 weeks in body weight	CFB to 56 weeks in body weight	CFB to 52 weeks in body weight	CFB to 26 weeks in body weight

Abbreviations: CFB, change from baseline; IPD MR, individual patient data meta-regression; OAD, oral antidiabetic drug; OD, once-daily; OW, once-weekly; s.c., subcutaneous.

*CFB to 52 weeks in HbA_1c_ (endpoint used in our analyses) was a secondary endpoint in PIONEER 2.

The rationale for selecting SUSTAIN 2, 3, and 8 was based on the similarities in patient selection criteria used (ie, background metformin) and in trial duration (52 to 56 weeks; Table 1) (8, 14, 15). The inclusion of patients receiving OW semaglutide on background metformin monotherapy from SUSTAIN 2 and 3 (trials of similar length as SUSTAIN 8, but with broader inclusion criteria in terms of background medication) increased the sample size and amount of data available for analysis. PIONEER 2 was selected for comparison because patients were on background metformin monotherapy, and this was the only Novo Nordisk-sponsored trial with available IPD for an SGLT-2i (OD empagliflozin) that had not already been compared with OW semaglutide directly in an RCT (9).

### Outcomes analyzed

The primary efficacy outcomes of interest in this analysis were change from baseline to end-of-treatment in HbA_1c_ (%-point) and body weight (kg). Responder outcomes were also analyzed, including the proportion of patients achieving HbA_1c_ targets (<7.0% and ≤6.5%), weight-loss responses (≥5% and ≥10%) and composite responder outcomes of: HbA_1c_ reduction ≥1.0% and weight loss ≥5%; HbA_1c_ <7.0% and weight loss ≥5%; HbA_1c_ <7.0% with no severe or blood glucose-confirmed hypoglycemia and no weight gain. Other clinically relevant measures of efficacy were analyzed, including change from baseline to end-of-treatment in body mass index (BMI), waist circumference, systolic and diastolic blood pressure, lipid parameters (total cholesterol, low-density lipoprotein cholesterol [LDL-C], high-density lipoprotein cholesterol [HDL-C] and triglycerides), and eGFR.

### Statistical analysis

As efficacy was the focus of these analyses, treatment effects were evaluated using data from all randomized patients (full analysis set [FAS]), prior to initiating rescue medication or discontinuation of treatment. For analysis of continuous variables, such as change from baseline in HbA_1c_, a mixed-effects model for repeated measurements (MMRM) regression was used to compare OW semaglutide and OD empagliflozin (16, 17). MMRM is a likelihood-based method that allows statistical inference when analyzing incomplete datasets, through imputation of missing values. Data missing from the period after initiating rescue medication or discontinuation of treatment were imputed using the MMRM with the same fixed effects and covariates as the meta-regression models, but within-trial (18). For dichotomous responder outcomes, such as patients achieving HbA_1c_ targets, a logistic regression model with logit link using data from the end-of-treatment visit was used. The responder outcome analyses used a combination of the observed and imputed values from the end-of-treatment visit to determine response.

Fixed and random effects (ie, baseline covariates) that could influence outcomes were explored. These can be broadly categorized into 2 groups: prognostic factors and effect modifiers. Prognostic factors affect outcomes independent of treatment, whereas effect modifiers affect outcomes that are dependent on treatment and the level of the variable (12). Prognostic factors and effect modifiers can be selected and included in statistical models to address variations in the trial/population resulting from the lack of randomization. In IPD meta-regression analyses, potential prognostic factors and effect modifiers can be adjusted at an individual patient level, allowing for a better isolation of the effect of a single factor (treatment) on an outcome of interest than might be achieved with published aggregate data, thereby enabling a potentially less-biased comparison between trials.

Through a combination of statistical analyses of data on file, consultation with clinical experts, and review of published literature, the potential prognostic factors and effect modifiers were identified and incorporated into each model in our analyses (Table 2). Although patient selection and duration were consistent across trials, we followed the recommendations for unanchored indirect comparison analyses provided by the *National Institute for Health and Care Excellence Decision Support Unit Technical Support Document 18* (12). These recommendations state that potential prognostic factors and effect modifiers should be determined a priori, starting with the identification of those factors of clinical relevance from published evidence (Table 2). The adjustment for potential prognostic factors and effect modifiers attempted to control for any differences in included treatment arms that might have impacted the estimated treatment effect.

**Table 2.** Potential Prognostic Factors and Effect Modifiers Included in Each of the IPD MR Analyses

Outcomes Analyzed	Potential Prognostic Factors and Effect Modifiers Included in Each Model									
	BL HbA_1c_ (PF and EM)	BL body weight (PF and EM)	T2D duration (PF and EM)	eGFR (EM)	Hypothyroidism (PF and EM)	Heart failure (PF)	Smoking status (PF and EM)	Age (PF and EM)	Sex (PF and EM)	Race (PF)
HbA_1c_ change from BL	X	X	X	X			X	X	X*	X
Body weight change from BL		X	X	X	X	X	X*	X^†^	X	X
HbA_1c_ responders	X		X	X			X	X	X*	X
Weight-loss responders		X	X	X	X	X	X*	X^†^	X	X
Composite responders	X	X	X	X	X	X	X	X	X	X
Other clinically relevant efficacy outcomes			X	X			X	X	X*	X

Published literature was available for BL HbA_1c_, BMI, T2D duration, and eGFR (19), hypothyroidism (20), heart failure (21-24), and smoking status (25-27). The baseline value for each outcome was used as a covariate. Composite responder analyses were HbA_1c_ reduction of ≥1.0% and weight-loss of ≥5%, HbA_1c_ <7.0% and weight-loss of ≥5%, and HbA_1c_ <7.0% with no severe or blood glucose–confirmed hypoglycemia and no weight gain. Other clinically relevant efficacy outcomes were change in BMI, waist circumference, diastolic blood pressure, systolic blood pressure, lipid parameters (total cholesterol, triglycerides, LDL-C, HDL-C) and eGFR.

Abbreviations: BL, baseline; BMI, body mass index; eGFR, estimated glomerular filtration rate; EM, potential effect modifier; HDL-C, high-density lipoprotein cholesterol; IPD MR, individual patient data meta-regression; LDL-C, low-density lipoprotein cholesterol; PF, prognostic factor; T2D, type 2 diabetes.

*PF only; ^†^EM only.

Potential prognostic factors and effect modifiers for HbA_1c_ and body weight analyses (change from baseline and responder analyses) are shown in Table 2. Other efficacy analyses generally used the same potential prognostic factors and effect modifiers as the primary HbA_1c_ analysis, except that the baseline value for each respective outcome was used as a covariate rather than HbA_1c_ at baseline. For composite responder outcome analyses, including HbA_1c_ targets and weight-loss responses, a combination of those factors from the separate HbA_1c_ and body weight analyses were used.

### Complementary analyses

Analyses of the primary outcomes unadjusted for potential prognostic factors and effect modifiers were also performed to assess the potential impact of these on the outcomes analyzed. In addition to the consideration of potential prognostic factors and effect modifiers, 2 complementary analyses were performed to assess the robustness of the findings from the primary analyses (change in HbA_1c_ and body weight). Complementary analysis 1 included all data on patients’ postbaseline measurements, up to and including the end-of-treatment visit from the in-trial observation period (ie, all randomized patients irrespective of treatment discontinuation or use of rescue medication), to investigate any potential impact of excluding observations of patients receiving rescue medication, or not receiving treatment, on treatment effects. Complementary analysis 2 included data from the SUSTAIN 8 and PIONEER 2 trials only. These 2 trials are very similar in design, duration of observation, use of background medication, and patient selection criteria (Table 1). The purpose of complementary analysis 2 was to evaluate whether inclusion of patients with a longer observation period for semaglutide than empagliflozin affected the results from the primary analyses (which included data from the SUSTAIN 2 and 3 trials of 56 weeks).

## Results

### Patient inclusion and baseline characteristics

**Figure 2.**
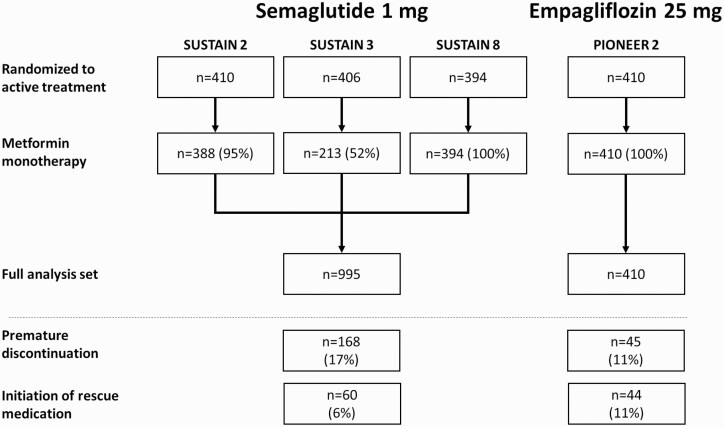
Disposition of patients inadequately controlled on metformin monotherapy included in the IPD MR. Abbreviation: IPD MR, individual patient data meta-regression.

In total, 995 patients on OW semaglutide 1 mg were included in the FAS for the primary efficacy analyses: 388 from SUSTAIN 2, 213 from SUSTAIN 3, and 394 patients from SUSTAIN 8 (all patients in the SUSTAIN 8 OW semaglutide arm; Fig. 2). In the OD empagliflozin 25 mg group, all 410 patients from PIONEER 2 receiving empagliflozin were included (Fig. 2). Patient baseline characteristics were similar between both groups and were similar across the 3 included SUSTAIN trials (Table 3). Of the 995 patients receiving OW semaglutide, 17% (n = 168) discontinued study medication and 6% (n = 60) initiated rescue medication, compared with 11% (n = 45) and 11% (n = 44), respectively, of patients on OD empagliflozin (Fig. 2).

**Table 3.** Baseline Patient Characteristics in SUSTAIN 2, 3, and 8, and PIONEER 2 Trials in the FAS of This IPD MR Analysis

	OW Subcutaneous Semaglutide 1 mg				OD Oral Empagliflozin 25 mg
Number of patients contributing to FAS per trial	SUSTAIN 2 n = 388	SUSTAIN 3 n = 213	SUSTAIN 8 n = 394	SUSTAIN pooled n = 995	PIONEER 2 n = 410
Age, years	56.0 (9.5)	55.0 (10.6)	55.7 (11.0)	55.6 (10.4)	57.8 (10.0)
Female, %	50.5	49.3	43.4	47.4	49.0
Race, %					
White	69.1	85.0	75.4	75.0	86.1
Black/African American	5.9	7.0	7.1	6.6	8.0
Asian	23.2	1.9	15.7	15.7	5.1
American Indian/Alaskan Native	0	0.9	0.3	0.3	0
Other*	1.8	5.2	1.5	2.4	0.7
BMI, kg/m^2^	32.6 (6.6)	34.3 (7.7)	32.2 (6.8)	32.8 (7.0)	32.8 (5.9)
Waist circumference, cm	106.7 (14.5)	111.8 (15.2)	106.5 (15.8)	107.7 (15.3)	108.5 (16.7)
Weight, kg	89.6 (20.7)	96.7 (23.0)	90.6 (22.6)	91.5 (22.1)	91.3 (20.1)
T2D duration, years	6.7 (5.6)	8.0 (5.5)	7.5 (5.9)	7.3 (5.7)	7.7 (6.3)
HbA_1c_, %	8.0 (0.9)	8.3 (1.0)	8.3 (1.0)	8.2 (1.0)	8.1 (0.9)
Fasting plasma glucose, mg/dL	167.3 (39.4)	190.3 (49.7)	168.6 (49.4)	172.8 (46.8)	174.0 (45.2)
Blood pressure, mmHg					
Diastolic blood pressure	81.0 (9.1)	80.3 (9.2)	78.9 (9.3)	80.0 (9.2)	80.0 (9.2)
Systolic blood pressure	132.9 (14.0)	132.0 (15.1)	129.4 (14.7)	131.3 (14.6)	131.9 (14.6)
Lipids, mg/dL					
Total cholesterol	185.9 (41.6)	192.5 (44.9)	176.3 (40.1)	183.45 (42.2)	183.9 (42.9)
LDL-C	103.2 (33.5)	106.9 (38.0)	97.6 (34.9)	101.75 (35.3)	102.95 (33.7)
HDL-C	47.9 (12.2)	47.6 (12.0)	44.9 (11.2)	46.65 (11.8)	45.8 (11.0)
Triglycerides	186.1 (144.7)	206.8 (154.4)	179.7 (124.2)	188.0 (139.35)	187.1 (127.1)
eGFR, mL/min/1.73m^2^	97.9 (14.2)	98.2 (15.4)	97.9 (16.5)	97.9 (15.4)	94.5 (15.1)
History of hypothyroidism, yes, %	5.2	9.9	7.9	7.2	9.8
History of heart failure, yes, %	6.2	2.3	1.3	3.4	3.9
Smoking status, %					
Current	12.9	16.4	14.2	14.2	14.4
Previous	24.2	30.5	30.7	28.1	30.0
Never	62.9	53.1	55.1	57.5	55.6

Data are mean (standard deviation) unless otherwise stated.

Abbreviations: BMI, body mass index; eGFR, estimated glomerular filtration rate (according to the Chronic Kidney Disease Epidemiology Collaboration equation); FAS, full analysis set; HDL-C, high-density lipoprotein cholesterol; IPD MR, individual patient data meta-regression; LDL-C, low-density lipoprotein cholesterol; OD, once-daily; OW, once-weekly; T2D, type 2 diabetes.

*Includes patients whose race was not available in study records.

In complementary analysis 1, OW semaglutide patient numbers were the same as for the primary analysis, whereas for complementary analysis 2, only the 394 patients from SUSTAIN 8 were included in the FAS (40% of the pooled number), of whom 16% (n = 62) discontinued study medication and 7% (n = 29) initiated rescue medication. The empagliflozin group remained unchanged from the primary analyses in both complementary analyses.

### Primary efficacy analyses

**Figure 3.**
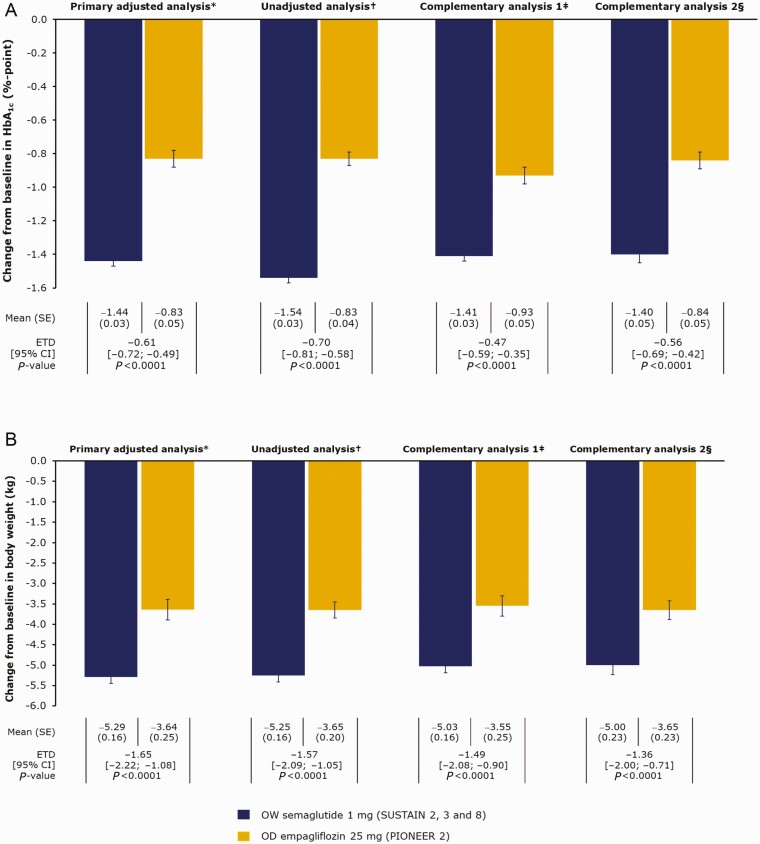
Change from baseline in a) HbA_1c_ and b) body weight with OW semaglutide 1 mg vs OD empagliflozin 25 mg at week 52. *The primary analysis used data from all randomized patients while on treatment without rescue medication, adjusted for potential prognostic factors and effect modifiers listed in Table 2. ^†^The unadjusted analysis used data from all randomized patients while on treatment without rescue medication, *not* adjusted for potential prognostic factors and effect modifiers listed in Table 2. ^‡^Complementary analysis 1 used “in-trial” data (data from all randomized patients irrespective of treatment discontinuation or use of rescue medication). ^§^Complementary analysis 2 included patient data from SUSTAIN 8 and PIONEER 2 only (both 52 weeks in duration; n = 394 for OW semaglutide). Abbreviations: CI, confidence interval; ETD, estimated treatment difference; OD, once-daily; OW, once-weekly; SE, standard error.

Our analyses showed that OW semaglutide 1 mg significantly lowered mean HbA_1c_ vs empagliflozin 25 mg, by 1.44%-point vs 0.83%-point, respectively (estimated treatment difference [ETD]: −0.61%-point [95% confidence interval (CI): −0.72; −0.49]; *P* < 0.0001; Fig. 3a). Our analyses also showed that OW semaglutide significantly reduced mean body weight vs OD empagliflozin: 5.29 kg vs 3.64 kg, respectively (ETD: −1.65 kg [95% CI: −2.22; −1.08]; *P* < 0.0001; Fig. 3b). When analyzed without inclusion of any potential prognostic factors and effect modifiers, these results remained numerically similar to the adjusted values (Fig. 3a and 3b).

Complementary analyses were consistent with the findings of the primary efficacy analyses, showing a significant benefit in favor of OW semaglutide vs OD empagliflozin. In complementary analysis 1 (using data from the in-trial observation period), the ETD was −0.47%-point (95% CI: −0.59; −0.35; *P* < 0.0001). In complementary analysis 2 (using only SUSTAIN 8 and PIONEER 2 data), the ETD was −0.56%-point (95% CI: −0.69; −0.42; *P* < 0.0001; Fig. 3a). Similarly, in complementary analysis 1 for body weight, the ETD was −1.49 kg (95% CI: −2.08; −0.90; *P* < 0.0001). In complementary analysis 2 for body weight, the ETD was −1.36 kg (95% CI: −2.00; −0.71; *P* < 0.0001; Fig. 3b).

### Responder outcomes analysis

Our analyses showed that a significantly greater proportion of patients on OW semaglutide vs OD empagliflozin achieved clinically relevant HbA_1c_ targets of <7.0% (69.1% vs 39.3%; odds ratio [OR]: 4.37 [95% CI: 3.33; 5.74]; *P* < 0.0001) and ≤6.5% (54.2% vs 19.0%; OR: 5.99 [95% CI: 4.46; 8.05]; *P* < 0.0001; Table 4). Similarly, a significantly greater proportion of patients on OW semaglutide vs OD empagliflozin achieved clinically relevant weight-loss responses of ≥5% (50.9% vs 37.1%; OR: 1.91 [95% CI: 1.50; 2.44]; *P* < 0.0001) and ≥10% (20.6% vs 7.3%; OR: 3.80 [95% CI: 2.53; 5.71]; *P* < 0.0001; Table 4).

**Table 4.** Proportion of Patients Achieving HbA_1c_ Targets and Weight-Loss Responses, and Composite Responder Outcomes

	OW Semaglutide 1 mg, n = 995 (SUSTAIN 2, 3, and 8)	OD Empagliflozin 25 mg, n = 410 (PIONEER 2)	Odds Ratio [95% CI]	*P* Value
**Responder analyses**				All <0.0001 in favor of semaglutide
Patients achieving HbA_1c_ targets, %				
<7.0%	69.1	39.3	4.37 [3.33; 5.74]	
≤6.5%	54.2	19.0	5.99 [4.46; 8.05]	
Patients achieving weight-loss responses, %				
≥5%	50.9	37.1	1.91 [1.50; 2.44]	
≥10%	20.6	7.3	3.80 [2.53; 5.71]	
**Composite responder analyses**				All <0.0001 in favor of semaglutide
Patient achieving HbA_1c_ reduction of ≥1.0% and weight-loss of ≥5%, %	41.5	16.8	3.73 [2.78; 5.00]	
Patients achieving HbA_1c_ <7.0% and weight-loss of ≥5%, %	43.8	19.0	3.67 [2.75; 4.90]	
Patients achieving HbA_1c_ <7.0% with no severe or blood glucose-confirmed hypoglycemia* and no weight gain, %	63.0	35.6	3.75 [2.87; 4.89]	

Data in table are model-based estimates, adjusted for potential prognostic factors and effect modifiers.

Abbreviations: ADA, American Diabetes Association; CI, confidence interval; OD, once-daily; OW, once-weekly.

*Defined as an episode of hypoglycemia that was severe according to the ADA classification (28) or confirmed by a glucose value <56 mg/dL, with symptoms consistent with hypoglycemia.

Our analyses also showed that a significantly greater proportion of patients on OW semaglutide than OD empagliflozin achieved the following composite responses: HbA_1c_ reduction of ≥1.0% and weight loss of ≥5%, HbA_1c_ <7.0% and weight loss of ≥5%, and HbA_1c_ <7.0% with no severe or blood glucose–confirmed hypoglycemia and no weight gain (all *P* < 0.0001; Table 4).

### Analysis of other clinically relevant measures of efficacy

Our analyses showed that OW semaglutide provided significantly greater reductions than OD empagliflozin in BMI, waist circumference, total cholesterol, LDL-C (all *P* < 0.0001) and triglycerides (*P* < 0.01), whereas OD empagliflozin provided significantly greater reductions than OW semaglutide in diastolic blood pressure (*P* < 0.05) and significantly greater increases in HDL-C (*P* < 0.01; Table 5). There was no significant difference between OW semaglutide and OD empagliflozin in regard to systolic blood pressure or change in eGFR.

**Table 5.** Change From Baseline in Other Clinically Relevant Efficacy Measures with OW Semaglutide 1 mg vs OD Empagliflozin 25 mg at Week 52

	OW Semaglutide 1 mg, n = 995 (SUSTAIN 2, 3, and 8)	OD Empagliflozin 25 mg, n = 410 (PIONEER 2)	Estimated Treatment Difference [95% CI]	*P* Value*
BMI, kg/m^2^	−1.92	−1.32	−0.60 [−0.81; −0.39]	<0.0001
Waist circumference, cm	−4.66	−2.76	−1.90 [−2.54; −1.26]	<0.0001
Blood pressure, mmHg				
Diastolic blood pressure	−1.27	−2.39	1.12 [0.27; 1.97]	0.0103
Systolic blood pressure	−4.11	−4.48	0.37 [−0.95; 1.68]	0.5842
Lipid parameters, mg/dL				
Total cholesterol	−6.15	4.14	−10.28 [−13.56; −7.01]	<0.0001
Triglycerides	−31.16	−15.13	−16.03 [−28.17; −3.90]	0.0097
LDL-C	−2.48	4.18	−6.66 [−9.44; −3.87]	<0.0001
HDL-C	1.53	2.63	−1.10 [−1.89; −0.30]	0.0073
eGFR, mL/min/1.73m^2^	0.15	−0.06	0.21 [−0.65; 1.07]	0.6304

Data in table are model-based estimates, adjusted for potential prognostic factors and effect modifiers.

Abbreviations: BMI, body mass index; CI, confidence interval; eGFR, estimated glomerular filtration rate; HDL-C, high-density lipoprotein cholesterol; LDL-C, low-density lipoprotein cholesterol; OD, once-daily; OW, once-weekly.

*All significant *P* values (*P* < 0.05) favor semaglutide, except diastolic blood pressure and HDL-C, which favor empagliflozin.

## Discussion

In this indirect IPD meta-regression analysis of patients with T2D whose disease was inadequately controlled on background metformin monotherapy, OW semaglutide 1 mg was superior to OD empagliflozin 25 mg in reducing HbA_1c_ and lowering body weight from baseline to end-of-treatment at ~1 year. Complementary analyses supported these findings. Our analyses also provide evidence that OW semaglutide may have superior efficacy vs OD empagliflozin across other clinically relevant measures, including BMI, waist circumference, total cholesterol, LDL-C, and triglyceride levels, as well as HbA_1c_ targets and weight-loss responses. Our analyses found that OD empagliflozin significantly reduced diastolic blood pressure and improved HDL-C vs OW semaglutide. There was no difference between OW semaglutide and OD empagliflozin with regards to systolic blood pressure or eGFR.

The findings of the primary analyses from this IPD meta-regression analysis are consistent with those previously reported by Sharma et al. in their NMA for a 26-week follow-up, in which there was indirect evidence that OW semaglutide 1 mg significantly reduced HbA_1c_ and body weight (Table 6) vs OD empagliflozin 25 mg (*P* < 0.05 for both) (13). However, comparisons across studies with different designs should be interpreted with caution. The findings of this IPD meta-regression analysis, which used data from SUSTAIN 2, 3, and 8 and PIONEER 2, are comparable with the magnitude of responses reported in the individual RCTs comparing a GLP-1RA with an SLGT-2i directly (SUSTAIN 8 and PIONEER 2; Table 6). We analyzed data from a 1-year period because it has been previously established that treatment differences, particularly changes in body weight, are not always consistent over time. For example, in SUSTAIN 8, OW semaglutide showed a nonlinear reduction in body weight in patients on background metformin monotherapy over the 52-week period (8). The data from this IPD meta-regression show that the difference in responses for change in HbA_1c_ and body weight reduction from baseline was significantly better at 1 year with OW semaglutide than with OD empagliflozin, adding to the existing evidence from the NMA of significant results at 26 weeks.

**Table 6.** Comparison of the Change in HbA_1c_ and Body Weight from Baseline in Direct and Indirect Comparisons of OW or Oral Semaglutide vs SGLT-2is

Mean absolute change in HbA_1c_ from baseline (%-point)			ETD
**IPD MR analysis** (52 weeks) All randomized patients on treatment without rescue medication, observed and imputed	OW semaglutide 1 mg	OD empagliflozin 25 mg	
	−1.44	−0.83	−0.61
**NMA** (26 ± 4 weeks) Mean differences of modeled change from baseline fixed effects	OW semaglutide 1 mg	OD empagliflozin 25 mg	
	NR*	NR*	−0.80^†^
	OW semaglutide 1 mg	OD canagliflozin 300 mg	
	NR*	NR*	−0.66^†^
**SUSTAIN 8** (52 weeks) All randomized patients on treatment without rescue medication, observed and imputed	OW semaglutide 1 mg	OD canagliflozin 300 mg	
	−1.5	−1.0	−0.49
**PIONEER 2** (52 weeks) Trial product estimand (on trial product without rescue medication)	OD oral semaglutide 14 mg	OD empagliflozin 25 mg	
	−1.3	−0.8	−0.5
Mean absolute change in body weight from baseline (kg)			ETD
**IPD MR analysis** (52 weeks) All randomized patients on treatment without rescue medication, observed and imputed	OW semaglutide 1 mg	OD empagliflozin 25 mg	
	−5.29	−3.64	−1.65
**NMA** (26 ± 4 weeks) Mean differences of modeled change from baseline fixed effects	OW semaglutide 1 mg	OD empagliflozin 25 mg	
	NR*	NR*	−2.05^†^
	OW semaglutide 1 mg	OD canagliflozin 300 mg	
	NR*	NR*	−1.59^†^
**SUSTAIN 8** (52 weeks) All randomized patients on treatment without rescue medication, observed and imputed	OW semaglutide 1 mg	OD canagliflozin 300 mg	
	−5.3	−4.2	−1.06
**PIONEER 2** (52 weeks) Trial product estimand (on trial product without rescue medication)	OD oral semaglutide 14 mg	OD empagliflozin 25 mg	
	−4.7	−3.8	−0.9

Abbreviations: ETD, estimated treatment difference; IPD MR, individual patient data meta-regression; NMA, network meta-analysis; NR, not reported; OD, once-daily; OW, once-weekly.

*Not reported as the NMA showed only ETD between OW semaglutide and OD empagliflozin, not the mean absolute change from baseline for each individual treatment. ^†^Placebo-corrected ETD (anchored comparison).

IPD meta-regression is among the most suitable options for indirect comparisons where the availability of data permits (10, 12). Furthermore, additional clinically relevant endpoints, such as BMI and lipid parameters, were analyzed in this IPD meta-regression, as they had not been assessed previously because of the absence of available published data (13). Data used in these analyses were obtained from well-designed RCTs that were similar in design and had similar patient eligibility criteria, and which captured key baseline characteristics; furthermore, each analysis used a large sample size to analyze the endpoints assessed in this IPD meta-regression. To reduce potential selection bias, all data from patients on a background of metformin monotherapy that were available at the time of analysis were included from the SUSTAIN trials with a duration of 1 year. Moreover, the use of potential prognostic factors and effect modifiers in regression analyses allowed adjustment for factors that were imbalanced between trials and which could potentially influence outcome, even if these imbalances were minor. Finally, the results from the complementary and unadjusted analyses were consistent with findings from the primary analyses, suggesting that the treatment effect observed in our analysis is unlikely to be confounded by the imputation of missing data, trial length, or patient characteristics. Complementary analysis 1, which included all data on patients’ postbaseline measurements irrespective of treatment discontinuation or use of rescue medication, suggested there was no bias in the parameter estimates as a consequence of imputing missing data. Complementary analysis 2, which included 2 trials of the same length, indicated no effect of trial length on outcomes.

Limitations of this indirect IPD meta-regression comparison include the unanchored analysis approach, in which the comparators analyzed were not assessed vs a common comparator in their individual trials. To compensate for this, potential prognostic factors and effect modifiers were selected according to statistical analyses, clinical expertise, and from published data (12). Unanchored indirect comparisons assume that all potential prognostic factors and effect modifiers are identified and there is no available published evidence on this population to confirm that all such potential prognostic factors and effect modifiers were included (12). Furthermore, although the trials were similar in design, there were some inter-trial differences. For example, SUSTAIN 2 and SUSTAIN 8 were double-blind trials, whereas SUSTAIN 3 and PIONEER 2 were open-label. In addition, SUSTAIN 2 and 3 were 56 weeks in duration, while SUSTAIN 8 and PIONEER 2 were 52 weeks. However, the complementary analysis including SUSTAIN 8 and PIONEER 2 only (identical trial length) was consistent with the findings of the primary analysis, suggesting that there is minimal impact of trial duration on our results. Moreover, the unadjusted analyses excluding potential prognostic factors and effect modifiers were consistent with the adjusted analyses, indicating a minimal confounding effect of patient characteristics on treatment difference. Finally, treatment decisions should involve a consideration of the trade-off between safety and efficacy, while these analyses are limited to comparisons of measures of efficacy. However, the safety profiles of each treatment are well-characterized in the individual trial publications (8, 9, 14, 15).

This indirect comparison suggests that OW semaglutide 1 mg provides superior reductions in HbA_1c_ and body weight, as well as across other measures of efficacy, including BMI, waist circumference, and some lipid parameters, vs OD empagliflozin 25 mg in patients with T2D when added to metformin monotherapy. The findings presented here, are in alignment with a previously published indirect comparison with shorter follow-up times, provide additional results on clinically relevant measures of efficacy not previously assessed and may also provide additional evidence to inform treatment decisions.

## Data Availability

All data generated or analyzed during this study are included in this published article or in the data repositories listed in References.

## Abbreviations

BMI: body mass index
eGFR: estimated glomerular filtration rate
ETD: estimated treatment difference
FAS: full analysis set
GLP-1RA: glucagon-like peptide-1 receptor agonist
HbA_1c_: glycated hemoglobin
HDL-C: high-density lipoprotein cholesterol
IPD: individual patient data
LDL-C: low-density lipoprotein cholesterol
MMRM: mixed-effects model for repeated measurements
NMA: network meta-analysis
OD: once-daily
OR: odds ratio
OW: once-weekly
RCT: randomized controlled trial
SGLT-2i: sodium–glucose co-transporter-2 inhibitor
T2D: type 2 diabetes

## Additional Information

***Current Affiliation:*** Anna Sandberg’s affiliation was Novo Nordisk during development of the manuscript, but is now Oncopeptides AB, Luntmakargatan 46, Stockholm, Sweden.

***Disclosure Summary:*** I.L. received grant support from Merck, Mylan, Novo Nordisk, Pfizer, and Sanofi, personal fees from AstraZeneca, Boehringer Ingelheim, Eli Lilly, Intarcia, Janssen, Mannkind, Novo Nordisk, Sanofi, TARGETPharma, and Valeritas; and nonfinancial support from AstraZeneca, Boehringer Ingelheim, Eli Lilly, Janssen, Novo Nordisk, Pfizer, and Sanofi. M.S.C. reports being an unpaid board member of the Association for the Study of Obesity (ASO) and a faculty member of the Primary Care Academy of Diabetes Specialists (PCADS), an expert advisor to The National Institute for Health and Care Excellence (NICE), a part-time Medical Director at LighterLife (a commercial weight-loss company), a partner at Clifton Medical Centre, and Director at Rotherham Institute for Obesity Weight Management, Ltd; he is an ad hoc Medical Advisor for McDonald’s UK and a professional advisor for the Obesity Empowerment Network; he also reports research income/support from Abbott, Boehringer Ingelheim/Lilly, GlaxoSmithKline, Janssen, Leo, LighterLife, Novartis, and Novo Nordisk; advisory board support from Boehringer Ingelheim/Lilly, LighterLife, and Novo Nordisk. A.-M.C. and P.J. are employees of Novo Nordisk and own stocks in the company. J.L. is an employee of Novo Nordisk. A.S. was an employee of Novo Nordisk during the development of this manuscript and owns stocks in Novo Nordisk. R.S. was employed as an agency worker for Novo Nordisk to perform the statistical analysis in this publication. A.P. received consultancy fees for the statistical analyses presented in this manuscript; she has also received personal fees from Novo Nordisk, Sanofi, and Ultragenyx.
